# Natural Crown Space Maintainer for Cervical Crown–Root Fracture in a Young Athlete

**DOI:** 10.1155/crid/8851093

**Published:** 2026-04-24

**Authors:** Oyku Peker Gonulal, Meryem Sahin, Onur Kesici

**Affiliations:** ^1^ Department of Pediatric Dentistry, Mersin University, Mersin, Türkiye, mersin.edu.tr

## Abstract

Repetitive traumatic injuries are frequently observed among children and adolescents, particularly in those engaged in contact sports, where the risk is significantly elevated. The loss of anterior teeth may adversely affect an individual’s psychological and social well‐being, making such injuries especially important in younger patients. Dental practitioners, and pediatric dentists in particular, often encounter crown–root fractures in their clinical practice. Fiber‐reinforced splinting systems have recently emerged as an alternative to traditional space maintainers in dental treatment. In this report, we describe the management of a 13‐year‐old male patient with a cervical crown–root fracture, in whom the natural crown was repositioned and stabilized using a fiber‐reinforced space maintainer.

## 1. Introduction

Traumatic injuries in childhood constitute not only a considerable threat to overall health but also an underrecognized public health concern [1]. In early life, oral trauma may result in discoloration, structural abnormalities, or even the loss of teeth, leading to significant long‐term complications [2]. Among these, the loss of anterior teeth is particularly impactful, as it can negatively influence a child’s psychological and social well‐being. Moreover, children today tend to be more aware of their appearance and may be especially sensitive to the esthetic consequences of dental trauma [3]. Dental injuries are commonly encountered in both children and adolescents, and establishing an accurate diagnosis followed by appropriate management is crucial for ensuring favorable long‐term outcomes [4]. Ongoing dental development further complicates treatment planning, as it increases the challenges associated with orthodontic, periodontal, and restorative procedures, particularly in cases involving complex crown–root fractures of anterior teeth in pediatric patients [5].

The loss of an anterior tooth may adversely influence speech, facial appearance, and psychological status. Therefore, restoring such defects is of critical importance in clinical practice. With recent developments in adhesive dentistry, fiber‐reinforced composite‐supported fixed prostheses have emerged as a promising alternative to conventional treatment approaches, offering advantages such as superior esthetics, minimally invasive application, lower cost, improved bonding performance, and reversibility [6]. Previous research has indicated that the incorporation of fibers into composite resins enhances their mechanical properties, particularly by increasing resistance to fracture through effective stress distribution and absorption within the material [7, 8]. In this regard, braided glass fiber systems such as Angelus Interlig provide a dependable option. These preimpregnated fibers help prevent crack propagation and redirect functional stresses, thereby reducing the load transmitted to the residual tooth structure. Additionally, their braided architecture promotes more uniform force distribution over a wider area, contributing to improved control of polymerization shrinkage and occlusal loading.

Accordingly, Interlig fibers contribute to a more cohesive interaction between enamel, dentin, and restorative composite, thereby enhancing durability and supporting favorable clinical outcomes [9]. Anterior tooth loss may arise from various causes, including traumatic injury, endodontic complications, or periodontal pathology, and is often perceived as an esthetic emergency by patients. In such cases, the primary objective of dental treatment is to re‐establish esthetics, followed by the restoration of function. When the natural crown is preserved and remains free of extensive caries, structural damage, or discoloration, it may be repurposed as a natural pontic in the fabrication of a provisional prosthesis [10].

The present case report describes a 13‐year‐old male patient who required esthetic rehabilitation after experiencing recurrent trauma following prior management of a complicated crown–root fracture.

## 2. Case Presentation

**Figure 1:**
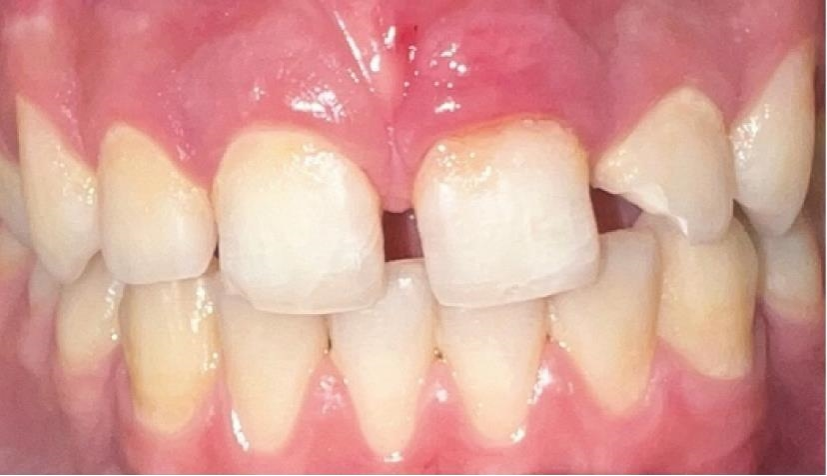
Preoperative intraoral view.

**Figure 2:**
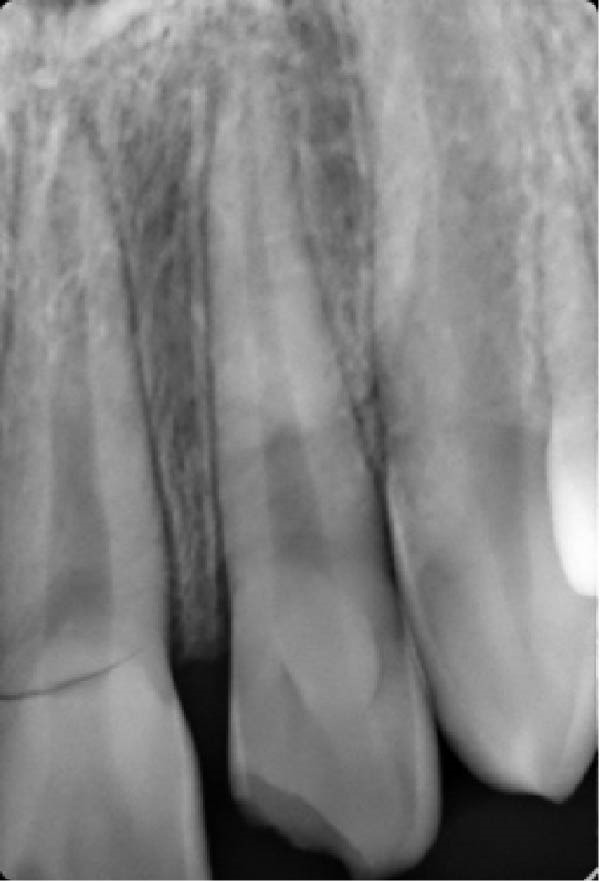
Preoperative periapical radiograph.

A 13‐year‐old male patient with an unremarkable medical history was admitted to the Department of Pediatric Dentistry at Mersin University 1 day after experiencing dental trauma during a wrestling activity. Clinical and radiographic assessments identified uncomplicated crown fractures in teeth #11 and #22, whereas tooth #21 demonstrated a complicated crown–root fracture accompanied by a mobile coronal fragment (Figures 1 and 2).

As an initial management step, the fractured surfaces of teeth #11 and #22 were temporarily restored using glass ionomer cement. The detached crown segment of tooth #21 was carefully removed in a single piece, and endodontic treatment was initiated for the remaining root portion.

The root canal procedure was completed in a single visit. Residual pulpal tissue within the separated crown fragment was eliminated, and the pulp chamber was sealed with a flowable composite material. After completion of the endodontic therapy, the canal orifice was protected using resin‐modified glass ionomer cement. Subsequently, the crown fragment was repositioned and adhesively reattached to the root using resin‐modified glass ionomer cement.

Teeth #13, #12, #11, #21, #22, and #23 were stabilized using a semi‐rigid wire splint. The patient was scheduled for follow‐up visits at 2 and 4 weeks, during which a noticeable reduction in tooth mobility was observed, permitting removal of the splint. Subsequent evaluations confirmed the absence of both mobility and clinical symptoms.

Nevertheless, 6 months later, the patient returned with a history of a second traumatic incident that had occurred 2 months earlier during a wrestling match, leading to the separation of the coronal portion of tooth #21. Clinical examination revealed that the root fragment had healed completely within the gingival tissue. The patient had retained the detached crown fragment in water.

Radiographic evaluation revealed no pathological findings, and the patient remained asymptomatic. However, loss of vitality was identified in tooth #11, indicating the need for root canal treatment, which was subsequently completed over two sessions.

**Figure 3:**
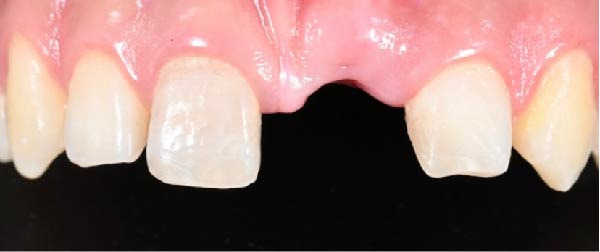
Aesthetic rehabilitation of teeth #11 and #22.

**Figure 4:**
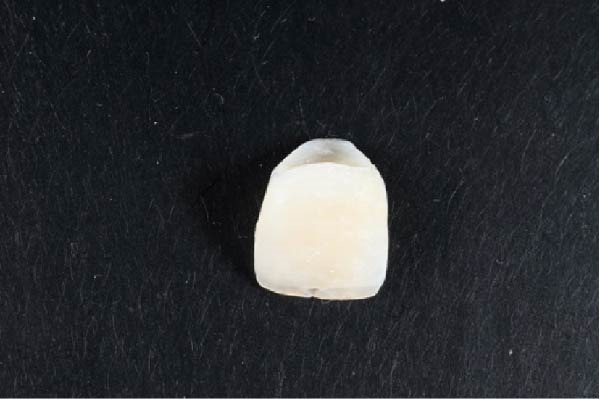
Recontouring of the crown of tooth #21 to harmonize with the gingival margin.

Esthetic rehabilitation of teeth #11 and #22 was carried out, and the crown of tooth #21 was recontoured to achieve harmony with the gingival margin (Figures 3 and 4).

**Figure 5:**
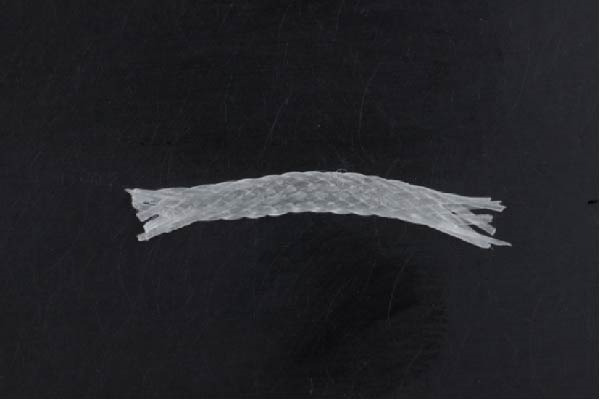
Woven glass fiber reinforcement system.

**Figure 6:**
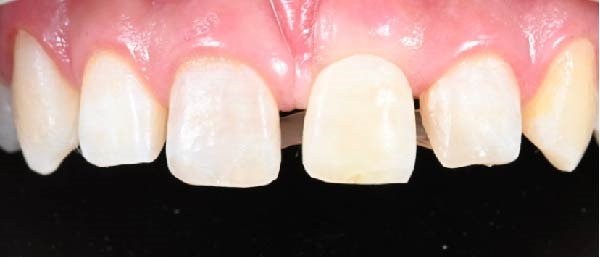
Postoperative intraoral view.

After isolating teeth #11 and #22, the palatal enamel surfaces were conditioned with 37% phosphoric acid (Panora 200, Imicryl, Turkey) for 20 s, gently rinsed, and air‐dried. A bonding agent (Ruby Bond, RubyDent, Istanbul, Turkey) was then applied, followed by placement of a flowable composite resin (T‐Com Flow, Nexobio, Korea). Subsequently, tooth #21 was positioned and secured using a woven glass fiber reinforcement system (Interlig, Angelus, Londrina, PR, Brazil) (Figure 5). The patient was scheduled for periodic follow‐up, and the esthetic outcome was considered satisfactory (Figure 6).

## 3. Discussion

Dental trauma represents one of the most frequently encountered emergency conditions in clinical dentistry [11] and often necessitates prompt management. Epidemiological data indicate that the global prevalence of traumatic dental injuries is ~15.2% in permanent dentition and 22.7% in primary dentition [12]. Children of school age are particularly vulnerable, as increased participation in physical activities and sports elevates the risk of falls and injuries. In preadolescent and adolescent populations, fractures are observed more commonly than avulsions, likely due to the greater density of the alveolar bone. The maxillary incisors are most commonly affected, especially in individuals presenting with increased overjet [13].

Dental trauma may lead to complicated crown–root fractures, typically described as oblique fractures extending in an apical and palatal direction, often involving pulp exposure [14]. Although traumatic dental injuries are more frequently seen in primary and mixed dentition, vertically oriented complicated crown–root fractures remain relatively uncommon in pediatric patients [15].

The management of complicated crown and crown–root fractures often necessitates a multidisciplinary approach, making treatment planning particularly demanding [16]. These cases are further complicated by the frequent subgingival extension of fracture margins, which limits the range of therapeutic options. Available treatment modalities include direct resin composite restorations, crown lengthening procedures, orthodontic or surgical extrusion, as well as reattachment of the fractured fragment [17].

Evidence from various clinical studies suggests that adhesive reattachment of the coronal fragment represents an effective and conservative treatment alternative for teeth with crown–root fractures [18, 19].

When replacing a missing maxillary anterior tooth, several factors must be taken into account, including the need for space maintenance, parental expectations, and the restoration of both function and esthetics [20].

In a study by Goel et al. [3], following the extraction of two primary incisors, restorative procedures were performed, and the extracted teeth were utilized as pontics supported by Interlig fiber. Although the buccal placement of the fiber was considered an esthetic limitation, this outcome was attributed to the challenges in patient cooperation due to the young age of the patient (5 years old).

Similarly, Javed et al. [6] reported a case in which a previously prepared pontic was fixed from the palatal aspect using Interlig fiber to replace a missing maxillary lateral incisor (tooth #12). The authors noted favorable esthetic and functional outcomes, with patient satisfaction maintained over a 2‐year follow‐up period.

In another study by Sheikh et al. [10], tooth #21 was extracted due to periodontal reasons and subsequently prepared for use as a pontic. After initial stabilization with Interlig fiber, additional reinforcement was achieved from the palatal side using orthodontic wire. The patient demonstrated satisfactory esthetic and functional results during a 1‐year follow‐up.

## 4. Conclusion

The replacement of missing anterior teeth plays a crucial role in improving patients’ self‐esteem and overall quality of life. Conventional fixed prosthetic approaches may not always be appropriate in every case, whereas removable appliances are frequently perceived as uncomfortable and impractical by patients. In this context, fiber‐reinforced prosthetic systems offer several advantages, including favorable esthetic outcomes, simplified laboratory procedures, and reduced clinical chair time.

The use of natural tooth structures as pontics further enhances both durability and esthetics, while also providing a suitable bonding substrate for composite materials. Therefore, fiber‐reinforced prostheses can be considered a reliable and effective treatment option for achieving both functional and esthetic rehabilitation in cases of anterior tooth loss.

## Author Contributions

**Oyku Peker Gonulal and Onur Kesici**: conceptualization, clinical procedure, data collection, literature review, writing – original draft, writing – review and editing. **Meryem Sahin**: clinical procedure, data collection – review and editing.

## Funding

The authors received no financial support for this study.

## Ethics Statement

Ethical approval for this study was obtained from the Clinical Research Ethics Committee of Mersin University.

## Consent

Written informed consent was obtained from the patient’s parents for the publication of this case report and the accompanying clinical images.

## Conflicts of Interest

The authors declare no conflicts of interest.

## Data Availability

The data that support the findings of this study are available from the corresponding author upon reasonable request.
